# Visceral Adiposity Index and Lipid Accumulation Product Index: Two Alternate Body Indices to Identify Chronic Kidney Disease among the Rural Population in Northeast China

**DOI:** 10.3390/ijerph13121231

**Published:** 2016-12-13

**Authors:** Dongxue Dai, Ye Chang, Yintao Chen, Shuang Chen, Shasha Yu, Xiaofan Guo, Yingxian Sun

**Affiliations:** Department of Cardiology, the First Hospital of China Medical University, 155 Nanjing North Street, Heping District, Shenyang 110001, China; 18240409506@163.com (D.D.) chang.ye@stu.xjtu.edu.cn (Y.C.); chenyintao1990@126.com (Y.C.); 13654004558@126.com (S.C.); yidasasa047717@hotmail.com (S.Y.); guoxiaofan1986@foxmail.com (X.G.)

**Keywords:** visceral adiposity index, lipid accumulation product index, chronic kidney disease, rural population

## Abstract

We aimed to compare the relative strength of the association between anthropometric obesity indices and chronic kidney disease (CKD). Another objective was to examine whether the visceral adiposity index (VAI) and lipid accumulation product index (LAPI) can identify CKD in the rural population of China. There were 5168 males and 6024 females involved in this cross-sectional study, and 237 participants (2.12%) suffered from CKD. Obesity indices included body mass index (BMI), waist circumference (WC), waist-to-height ratio (WHtR), VAI and LAPI. VAI and LAPI were calculated with triglyceride (TG), high-density lipoprotein (HDL), BMI and WC. VAI = [WC/39.68 + (1.88 × BMI)] × (TG /1.03) × (1.31/ HDL) for males; VAI = [WC/36.58 + (1.89 × BMI)] × (TG/0.81) × (1.52/HDL) for females. LAPI = (WC-65) × TG for males, LAPI = (WC-58) × TG for females. CKD was defined as an estimated glomerular filtration rate (eGFR) of less than 60 mL/min per 1.73 m^2^. The prevalence of CKD increased across quartiles for WHtR, VAI and LAPI. A multivariate logistic regression analysis of the presence of CKD for the highest quartile vs. the lowest quartile of each anthropometric measure showed that the VAI was the best predictor of CKD in females (OR: 4.21, 95% CI: 2.09–8.47, *p* < 0.001). VAI showed the highest AUC for CKD (AUC: 0.68, 95% CI: 0.65–0.72) and LAPI came second (AUC: 0.66, 95% CI: 0.61–0.70) in females compared with BMI (both *p*-values < 0.001). However, compared with the traditional index of the BMI, the anthropometric measures VAI, LAPI, WC, and WHtR had no statistically significant capacity to predict CKD in males. Our results showed that both VAI and LAPI were significantly associated with CKD in the rural population of northeast China. Furthermore, VAI and LAPI were superior to BMI, WC and WHtR for predicting CKD only in females.

## 1. Introduction

Chronic kidney disease (CKD) is a major worldwide public health issue that increases cardiovascular morbidity and mortality [1]. Recently, a high-fat, protein-rich, and high-carbohydrate diet became popular and, along with an improvement in living conditions, has increased the risk of impaired kidney function and CKD [2,3]. Some recent surveys have shown dietary factors may play a role in decreasing the prevalence of CKD in some countries [4]. Therefore, prevention and management of CKD seem to be particularly important. Obesity as a focus of growing attention has been associated with CKD, even in the early stage of an impaired kidney [5,6,7]. Classical anthropometric index the body mass index (BMI) has been widely used in obesity studies because of its recognizability [8]. For convenience and briefness, some authors also proposed other alternative obesity indices such as the waist-to-hip ratio (WHtR) and waist circumference (WC) to predict CKD [9,10].

The relationship between obesity and CKD is complex and not yet fully understood. Which anthropometric index is a better predictor for CKD is still a controversy. Previous studies showed a positive relationship between BMI and CKD [9,10]. The Framingham Heart Study conducted a 20-year follow-up study and indicated that BMI, as a representative of obesity, was associated with an increased risk of developing stage 3 CKD [11]. However, a Korean cohort study showed WC, not BMI, could predict a faster decline in renal function [12]. Moreover, some authors prefer WC while others support WHtR as a determinant of the risk of CKD [13,14]. Recently, some authors found gender-specific associations between obesity and CKD and proposed a conclusion that BMI, WC and WHtR increased the risk of CKD in diabetic or hypertensive male subjects, but all these indices were limited to screening for the CKD [15,16,17].

Moreover, two newer obesity indices were proposed. The visceral adiposity index (VAI) was found to increase cardiovascular risks in hypertension [18], left ventricular hypertrophy [19], diabetes [20], and CKD [21], and can even be associated with inflammation [22]. The lipid accumulation product index (LAPI) based on a combination of WC and the fasting concentration of circulating TG has been shown to predict the incident of diabetes [23] and the increased risk of CVD [24] and influenced all-cause mortality [24,25]. However, there has not been relative research conducted about the relationship between LAPI and CKD. Additionally, we do not know whether these two anthropometric measures containing blood lipids could outperform conventional indices in predicting CKD in the rural population.

We conducted this cross-sectional study in a general population to assess the capacity of these anthropometric measurements (VAI and LAPI) to identify individuals with CKD in the rural population of northeast China. We also compared the attributes of five anthropometric measurements (VAI, LAPI, BMI, WC, and WHtR), and attempted to determine whether the VAI and LAPI were superior to measurements of BMI, WC and WHtR.

## 2. Materials and Methods 

### 2.1. Study Population

This cross sectional survey was conducted from January 2012 to August 2013 in Liaoning Province, located in northeast China. A representative sample of individuals aged ≥35 years was selected to participate in assessing two new obesity indices (VAI and LAPI) for purposes of identifying cases of CKD in rural northeast China. Participants were selected using a multi-stage stratified random cluster sampling method. Step one, three counties (Dawa, Zhangwu, and Liaoyang County) were selected randomly from the rural areas of Liaoning province; step two, one town was randomly selected from each of the three counties. Step three, 26 rural villages in three towns were randomly selected for inclusion in the study. Participants who were pregnant, or had a malignant tumor or mental disorder were excluded. Finally, 11,192 participants (5168 males and 6024 females) were eligible for the study. The study protocol was approved by the Ethics Committee of China Medical University (Shenyang, China, ethic approved project identification code: 2011-2-2), and all procedures were performed in accordance with good ethical standards.

### 2.2. Data Collection

Data were collected during a single clinical visit by cardiologists and trained nurses using a standard questionnaire by face-to-face interview. Prior to conducting the survey, all eligible investigators were invited to attend a training and those who obtained a perfect score on a training test were allowed to participate this study. Additionally, the investigators received further instructions and support during data collection.

Data on age, sex and personal history (coronary artery disease, hypertension and diabetes) and details about lifestyle (current or past cigarette smoking, alcohol intake, diet habits, educational status and physical activity) were obtained through questionnaires.

### 2.3. Physical Measures

Blood pressure (BP) was measured using a standardized automatic electronic sphygmomanometer (HEM-907; Omron, Kyoto, Japan) in the sitting position after resting for at least 5 min, according to the recommended American Heart Association protocol. The average BP value was calculated after three different consecutive measurements. The participants were advised to avoid caffeinated beverages and exercise for at least 30 min before the measurement. During the measurement, each participant was seated with their tested arm supported at the level of the heart.

Weight and height were measured to the nearest 0.1 kg and 0.1 cm, respectively, with the participants wearing light-weight clothing and without shoes. WC was measured at the umbilicus using a non-elastic tape (to the nearest 0.1 cm), with the participants standing at the end of normal expiration. BMI was calculated as the individual’s weight in kilograms divided by the square of the height in meters. WHtR was calculated by dividing WC by height.

### 2.4. Laboratory Assays

A fasting blood sample was collected from each participant in the morning after at least 12 h of fasting. Blood samples were obtained from an antecubital vein and collected in Vacutainer^®^ tubes containing EDTA. Values for fasting plasma glucose (FPG), total cholesterol (TC), low-density lipoprotein cholesterol (LDL-C), high-density lipoprotein cholesterol (HDL-C), triglyceride (TG), and other routine blood biochemical indexes were obtained using an autoanalyzer. Serum creatinine (SCr) was measured enzymatically on an autoanalyzer. 

### 2.5. Definition of VAI and LAPI Score

VAI was calculated using the following formula [26]: Males: VAI = [WC/39.68 + (1.88 × BMI)] × (TG/1.03) × (1.31/HDL); Females: VAI = [WC/36.58 + (1.89 × BMI)] × (TG/0.81) × (1.52/HDL).

LAPI was calculated using the following formula [27]: Males: LAPI = (WC-65) × TG; Females: LAPI = (WC-58) × TG.

### 2.6. Definition of CKD

The estimated glomerular filtration rate (eGFR) was using the equation originating from the CKD Epidemiology Collaboration (CKD-EPI) equation [28]. For practical purposes, CKD was defined as an eGFR of less than 60 mL/min per 1.73 m^2^ [29].

### 2.7. Statistical Analysis

Descriptive statistics were calculated for all the variables, including continuous variables (reported as mean values and standard deviations) and categorical variables (reported as numbers and percentages). The differences between groups were evaluated using ANOVA (LSD) for continuous data and Chi-square test for categorical data. Quartiles of BMI, WC and WHtR were created and the prevalence of CKD was calculated in each quartile. Since VAI and LAPI were strongly correlated with sex, VAI and LAPI quartiles were determined for males and females separately. The odds ratios (ORs) and their 95% confidence intervals (CIs) for the presence of CKD were compared using the highest to the lowest quartile of each anthropometric index, and were estimated by logistic regression analysis with adjustments made for age, race, family income, education, smoking, and alcohol status. We used the area under the receiver-operating characteristic curve (AUC) and 95% CIs to assess the discriminatory power of each anthropometric index to assess the risk for CKD. All the statistical analyses were performed using SPSS version 22.0 software (SPSS Inc., Chicago, IL, USA) and *p*-values less than 0.05 were considered statistically significant.

## 3. Results

A total of 11,192 subjects (5168 males and 6024 females) aged ≥35 years participated in the study. There were 237 participants suffering from CKD and the prevalence of CKD was 2.12% among the rural populations in northeast China.

Table 1 presents the clinical and demographic characteristics of participants according to CKD. The average age was 53.83 ± 10.55, and subjects with CKD were significantly older (68.57 ± 9.45 years). Overall, the general population had low educational levels and family incomes in the rural areas. Additionally, almost all participants were of Han nationality. In females, all of the anthropometric indices except for BMI were significantly larger in subjects with CKD than in subjects without CKD. Furthermore, significant differences were found in systolic blood pressure, diastolic blood pressure, uric acid, TC, TG, LDL-C and FPG. Unexpectedly, current smoking and alcohol consumption were very low in the CKD group, maybe because they quit smoking and drinking after being diagnosed with CKD.

Table 2 shows the prevalence of CKD in quartiles of VAI, LAPI, BMI, WC and WHtR. The prevalence of CKD increased per quartile for all five anthropometric indices (first quartile vs. fourth quartile) in both sexes: VAI 0.5% vs. 2.3% for males, VAI 0.7% vs. 4.6% for females; LAPI 0.5% vs. 1.9% for males, LAPI 1.1% vs. 4.7% for females; BMI 0.9% vs. 1.8% for males, BMI 2.6% vs. 2.9% for females; WC 1.0% vs. 2.1% for males, WC 1.6% vs. 3.7% for females; WHtR 0.4% vs. 2.4% for males, WHtR 1.3% vs. 4.4% for females (all *p* < 0.05).

Table 3 shows the multiple logistic regression analysis results for each anthropometric index. After adjustment for age, race, family income, education, smoking, alcohol status, systolic and diastolic blood pressure, the ORs of CKD increased with the increasing quartiles for VAI. Considering the significant ORs of the presence of CKD for the four quartiles, the VAI was the best predictor of CKD. For the highest quartile vs. the lowest quartile of each anthropometric measure, VAI (OR: 4.80, 95% CI: 1.94–11.92 in males; OR: 4.21, 95% CI: 2.09–8.47 in females, both *p* < 0.001) had higher ORs than LAPI (OR: 3.58, 95% CI: 1.49–9.07 in males, *p* < 0.01; OR: 3.10, 95% CI: 1.71–5.61 in females, *p* < 0.001), BMI (OR: 2.27, 95% CI: 1.06–4.82 in males; OR: 1.80, 95% CI: 1.04–3.10 in females, both *p* < 0.05), WC (no significance in males; OR: 2.12, 95% CI: 1.25–3.58 in females, *p* < 0.01) and WHtR (OR: 3.20, 95% CI: 1.28–7.95 in males; OR: 1.87, 95% CI: 1.07–3.25 in females, both *p* < 0.05). 

**Figure 1:**
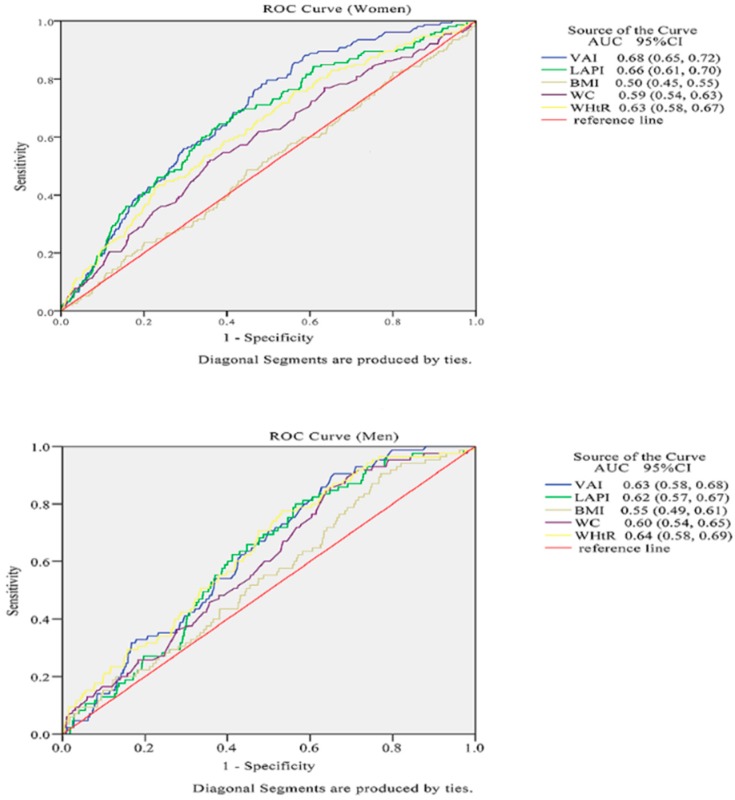
The discriminatory power of VAI, LAPI, BMI, WC and WHtR between subjects with or without CKD. Area under the receiver operating characteristic curve of VAI, LAPI, BMI, WC and WHtR to identify subjects with CKD according to sex.

Table 4 and Figure 1 show the AUC scores (and 95% CIs) of anthropometric measures in the prediction of CKD, and *p*-values were obtained from the respective comparisons between BMI and other anthropometric measures. VAI and LAPI were more significantly capable of predicting CKD in females than conventional indices. VAI had the highest AUC for CKD (AUC: 0.68, 95% CI: 0.65–0.72), LAPI was right behind it (AUC: 0.66, 95% CI: 0.61–0.70), and the BMI showed the lowest AUC value for CKD in females (AUC: 0.50, 95% CI: 0.45–0.55). However, compared with the traditional index of the BMI, other anthropometric indices had no statistically significant capacity to predict CKD in males.

## 4. Discussion

The main finding of this cross-sectional study was that all anthropometric indices (VAI, LAPI, BMI, WC, and WHtR) were significantly associated with CKD (eGFR < 60 mL/min per 1.73 m^2^) among rural populations in northeast China. To our knowledge, there has been no relevant literature confirming a significant association between LAPI and CKD so far. Our study has found for the first time that the LAPI had a positive correlation with CKD. Moreover, there are few relevant studies to compare the relative strength of the association between comprehensive obesity measurements and CKD, and the predictive power of traditional indices for chronic diseases is still controversial. Our study showed for the first time that VAI and LAPI had superior predictive ability for identifying CKD as compared to traditional indices in women, and BMI did not effectively predict the existence of CKD. Neither VAI nor LAPI had obvious advantageous capabilities for identifying the association between obesity and CKD compared to BMI in the male gender, while our study indicated that the two surrogate anthropometric indices VAI and LAPI were more suitable for identifying CKD in women.

Although there were several epidemiologic studies showing that obesity and CKD have increased in parallel worldwide and are positively correlated [30], the mechanisms by which changes in adiposity affect CKD are not entirely clear. Henegar JR and his colleagues conducted force-fed dog studies which showed a significant increase in kidney weight, plasma renin activity and insulin concentrations in obese dogs compared with lean dogs. These early hemodynamic and structural changes in kidney function could be influenced by a high-fat diet [31]. Moreover, some recent works proposed that obesity-related subclinical inflammation and oxidative stress could precede some changes in kidney function and structure such as increased albuminuria, and then induce renal damage [32,33]. As mentioned above, these potential mechanisms might give reasonable explanations as to why individuals with higher VAI or LAPI could have a higher risk of CKD in our study. As traditional predictive indices of obesity, BMI and WC predicting for CKD were relatively unhelpful among the rural population in northeast China because of the lower means of height, BMI, and WC than in Euramerican studies.

Some studies have pointed out that women were more likely to have abdominal obesity [34,35]. Furthermore, according to research on the southern Chinese population, Jun et al. found a strong correlation between VAI scores and CKD in females but not in males [34]. In the present study, as representative of visceral obesity, VAI and LAPI predicting for CKD seemed to be more valuable in females. The mechanisms underlying gender-specific differences are not known. Different sex hormones might act on the fat distribution which subsequently affects the association between obesity and CKD. However, Palmer BF conducted a review showing that since estrogens could regulate adipose deposition and function, males and postmenopausal females tended to deposit more visceral fat, while premenopausal females accrued subcutaneous fat, leading to the classic body shape which has been highly correlated to an increased chronic disease risk [36]. Animal studies have demonstrated that estrogen exerts renoprotective effects after whole-body acute alternatives of hemodynamics and estrogen doses improved the renal outcome in male rats [37]. In contrast, women had higher values of VAI, LAPI and WHtR compared with men in our study population. This discrepant phenomenon is probably attributed to ethnic differences and lifestyles. In rural areas of northeast China, the majority of Han people still maintain the traditional male-breadwinner model and the burden of low income has had males alternately engaging in agricultural and industrial activities. The activity intensity was great enough that males tended to have a lower distribution of abdominal fat compared to females of the same ages who were less active.

The recognition of the importance of visceral obesity has attained increasing attention in recent years. Someone proposed that imaging technologies such as CT and MRI should be applied to evaluate visceral adiposity because of their accuracy [38]. However, using them to screen for CKD in a large-scale population is hard to achieve due to the huge spending and high radiation exposure. VAI and LAPI have the advantages of low cost, easy measurements, and good social profit. Furthermore, research has shown VAI and LAPI are effective markers for identifying metabolic obesity [35,39] and correlate with metabolic syndrome [40]. We suggest that VAI and LAPI as effective markers of visceral obesity are simpler and more sensitive predictors of CKD than the traditional indices in females.

There are several limitations in our study. Firstly, the prevalence of CKD in our study was lower than the data from a Chinese national sample survey (10.8%) [41], and our population consisted mostly of peasants who had lots of physical activity. Therefore, the study was not nationally representative. Secondly, due to the relatively large sample size, we did not measure microalbuminuria/proteinuria and only used eGFR as the criterion for judging for impaired kidneys, which may result in ignorance of the CKD subjects with a normal GFR. Lastly, the study was a cross-sectional design which is unable to distinguish between cause and effect, and further exploration is needed.

## 5. Conclusions

Our present study found that VAI, LAPI, BMI, WC and WHtR were all significantly associated with CKD in both female and male genders after adjusting for confounding variables. In addition, our study indicated that VAI and LAPI showed a superior predictive ability for identifying CKD compared to conventional indices only in females.

## Figures and Tables

**Table 1:** Baseline characteristics of the study population.

Variables	Total (N = 11192)	CKD (n = 237)	Non-CKD (n = 10955)	*p*-Value
Age (years)	53.83 ± 10.55	68.57 ± 9.45	53.51 ± 10.35	<0.001
Males (%)	5168 (46.2)	85 (35.9)	5083 (46.4)	0.001
Education				<0.001
Low	5572 (49.8)	184 (77.6)	5388 (49.2)	
Middle	4569 (40.8)	44 (18.6)	4525 (41.3)	
High	1051 (9.4)	9 (3.8)	1042 (9.5)	
Family income (CNY/year)				<0.001
<5000	1382 (12.3)	64 (27.0)	1318 (12.0)	
5000–20,000	6108 (54.6)	119 (50.2)	5989 (54.7)	
>20,000	3702 (33.1)	54 (22.8)	3648 (33.3)	
Smokers (%)	3960 (35.4)	70 (29.5)	3890 (35.5)	0.057
Drinkers (%)	2524 (22.6)	15 (6.3)	2509 (22.9)	<0.001
Race				0.067
Han (%)	10618 (94.9)	231 (97.5)	10387 (94.8)	
Others ^a^ (%)	574 (5.1)	6 (2.5)	568 (5.2)	
Anthropometric measures				
BMI (kg/m^2^)				
Males	24.73 ± 3.55	25.41 ± 3.62	24.72 ± 3.54	0.075
Females	24.85 ± 3.75	24.80 ± 3.92	24.85 ± 3.75	0.858
WHtR				
Males	0.50 ± 0.06	0.53 ± 0.06	0.50 ± 0.06	<0.001
Females	0.52 ± 0.06	0.55 ± 0.07	0.52 ± 0.06	<0.001
WC (cm)				
Males	83.77 ± 9.74	87.29 ± 10.14	83.71 ± 9.72	0.001
Females	81.23 ± 9.70	84.04 ± 10.33	81.16 ± 9.68	<0.001
VAI				
Males	1.73 ± 2.20	2.06 ± 1.65	1.72 ± 2.21	0.187
Females	2.36 ± 2.40	3.37 ± 2.76	2.33 ± 2.38	<0.001
LAPI (cm·mmol/L)				
Males	35.53 ± 49.12	42.50 ± 37.36	35.41 ± 49.29	0.156
Females	40.94 ± 45.25	64.10 ± 80.61	40.34 ± 43.81	<0.001
Measurement indicators				
Uric acid (mg/dL)	291.84 ± 84.76	392.82 ± 112.57	289.66 ± 82.71	<0.001
SBP (mmHg)	141.67 ± 23.43	156.54 ± 26.36	141.35 ± 23.26	<0.001
DBP (mmHg)	82.03 ± 11.79	84.99 ± 15.72	81.97 ± 11.68	<0.001
LDL-c (mmol/L)	2.93 ± 0.82	3.26 ± 1.10	2.92 ± 0.81	<0.001
HDL-c (mmol/L)	1.41 ± 0.38	1.30 ± 0.37	1.41 ± 0.38	<0.001
TG (mmol/L)	1.64 ± 1.50	2.11 ± 1.71	1.63 ± 1.50	<0.001
TC (mmol/L)	5.24 ± 1.09	5.79 ± 1.65	5.22 ± 1.07	<0.001
FPG (mmol/L)	5.91 ± 1.64	6.58 ± 2.39	5.89 ± 1.62	<0.001

Abbreviations: VAI visceral adiposity index, LAPI lipid accumulation product index, BMI body mass index, WC waist circumference, WHtR waist-to-height ratio, CNY China Yuan (1 CNY = 0.145 USD), ^a^ including some ethnic minorities in China, such as Mongol and Manchu, DBP diastolic blood pressure, SBP systolic blood pressure, HDL-C high-density lipoprotein cholesterol, LDL-C low-density lipoprotein cholesterol, TC total cholesterol, TG triacylglycerol, FPG fasting plasma glucose.

**Table 2:** Prevalence of CKD in quartiles of VAI, LAPI, BMI, WC and WHtR.

Quartile (Males)	VAI	LAPI	BMI	WC	WHtR
1 (n of CKD [%])	6 (0.5)	6 (0.5)	12 (0.9)	22 (1.0)	6 (0.4)
2 (n of CKD [%])	20 (1.6)	20 (1.6)	26 (2.0)	12 (2.9)	13 (1.3)
3 (n of CKD [%])	29 (2.3)	35 (2.7)	24 (1.9)	21 (1.8)	37 (2.3)
4 (n of CKD [%])	30 (2.3)	24 (1.9)	23 (1.8)	30 (2.1)	29 (2.4)
**Quartile (Females)**	**VAI**	**LAPI**	**BMI**	**WC**	**WHtR**
1 (n of CKD [%])	10 (0.7)	16 (1.1)	39 (2.6)	25 (1.6)	19 (1.3)
2 (n of CKD [%])	23 (1.5)	28 (1.8)	35 (2.3)	33 (2.1)	25 (1.9)
3 (n of CKD [%])	50 (3.4)	38 (2.5)	48 (2.5)	39 (2.6)	42 (2.4)
4 (n of CKD [%])	69 (4.6)	70 (4.7)	30 (2.9)	55 (3.7)	66 (4.4)

Data presented as number (proportion); Abbreviations: VAI visceral adiposity index, LAPI lipid accumulation product index, BMI body mass index, CKD chronic kidney disease, WC waist circumference, WHtR waist-to-height ratio.

**Table 3:** Odds ratio (95% CI) of the presence of CKD for each anthropometric measure ^a^.

Quartile (Males)	VAI	LAPI	BMI	WC	WHtR
1 (reference)	1	1	1	1	1
2	2.59 [1.02, 6.61] *	2.65 [1.04, 6.76] *	2.65 [1.28, 5.47] **	2.74 [1.29, 5.83] **	2.83 [1.05, 7.63] *
3	3.87 [1.57, 9.56] **	4.86 [1.99, 11.85] **	2.26 [1.08, 4.72] *	1.52 [0.81, 2.86]	4.41 [1.82, 10.69] **
4	4.80 [1.94, 11.92] ***	3.58 [1.41, 9.07] **	2.27 [1.06, 4.82] *	1.75 [0.97, 3.15]	3.20 [1.28, 7.95] *
**Quartile (Females)**	**VAI**	**LAPI**	**BMI**	**WC**	**WHtR**
1 (reference)	1	1	1	1	1
2	1.71 [0.78, 3.71] *	1.59 [0.83, 3.06]	1.21 [0.73, 2.00]	1.63 [0.93, 2.86]	1.57 [0.83, 2.97]
3	3.63 [1.77, 7.44] ***	1.81 [0.96, 3.39]	1.40 [0.87, 2.25]	1.70 [0.99, 2.93]	1.67 [0.94, 2.98]
4	4.21 [2.09, 8.47] ***	3.10 [1.71, 5.61] ***	1.80 [1.04, 3.10] *	2.12 [1.25, 3.58] **	1.87 [1.07, 3.25] *

The between-cut points are 0.69, 1.13, and 1.98 for VAI (males); 1.06, 1.70, and 2.81 for VAI (females); 10.78, 21.96 and 43.33 for LAPI (males); 16.12, 28.18 and 50.83 for LAPI (females); 22.26, 24.46 and 26.87 for BMI (males); 22.22, 24.65 and 27.18 for BMI (females); 77.00, 83.10 and 90.00 for WC (males); 74.50, 81.00 and 87.30 for WC (females); 0.46, 0.50 and 0.54 WHtR (males); 0.48, 0.52 and 0.56 for WHtR (females); Abbreviations: VAI visceral adiposity index, LAPI lipid accumulation product index, BMI body mass index, CKD chronic kidney disease, WC waist circumference, WHtR waist-to-height ratio; * *p* < 0.05, ** *p* < 0.01, *** *p* < 0.001; ^a^ Adjusted for age, race, family income, education, smoking, alcohol status, systolic and diastolic blood pressure.

**Table 4:** The area under the curve of each anthropometric measure for the presence of CKD in both genders.

Index	Males (n = 5168)	Females (n = 6024 )
VAI	0.63 (0.58, 0.68)	0.68 (0.65, 0.72) ***
LAPI	0.62 (0.57, 0.67)	0.66 (0.61, 0.70) ***
BMI	0.55 (0.49, 0.61)	0.50 (0.45, 0.55) *
WC	0.60 (0.54, 0.65)	0.59 (0.54, 0.63) **
WHtR	0.64 (0.58, 0.69)	0.63 (0.58, 0.67)

Abbreviations: VAI visceral adiposity index, LAPI lipid accumulation product Index, BMI body mass index, CKD chronic kidney disease, WC waist circumference, WHtR waist-to-height ratio; * *p* < 0.05, ** *p* < 0.01, *** *p* < 0.001.

## References

[1] Matsushita K., Ballew S.H., Coresh J. (2016). Cardiovascular risk prediction in people with chronic kidney disease. Curr. Opin Nephrol. Hypertens..

[2] Okreglicka K. (2015). Health effects of changes in the structure of dietary macronutrients intake in western societies. Roczniki Panstwowego Zakladu Higieny.

[3] Odermatt A. (2011). The Western-style diet: A major risk factor for impaired kidney function and chronic kidney disease. Am. J. Physiol. Ren. Physiol..

[4] De Nicola L., Minutolo R. (2016). Worldwide growing epidemic of CKD: Fact or fiction?. Kidney Int..

[5] Jung C.H., Lee M.J., Kang Y.M., Hwang J.Y., Kim E.H., Park J.Y., Kim H.K., Lee W.J. (2015). The risk of chronic kidney disease in a metabolically healthy obese population. Kidney Int..

[6] Yu S., Yang H., Guo X., Zheng L., Sun Y. (2016). Association between obese phenotype and mildly reduced eGFR among the general population from rural northeast China. Int. J. Environ. Res. Public Health.

[7] Stepien M., Stepien A., Wlazel R.N., Paradowski M., Banach M., Rysz M., Rysz J. (2013). Obesity indices and adipokines in non-diabetic obese patients with early stages of chronic kidney disease. Med. Sci. Monit. Int. Med. J. Exp. Clin. Res..

[8] World Health Organization Expert Consultation. (2004). Appropriate body-mass index for Asian populations and its implications for policy and intervention strategies. Lancet.

[9] Wickman C., Kramer H. (2013). Obesity and kidney disease: Potential mechanisms. Semin. Nephrol..

[10] Gerchman F., Tong J., Utzschneider K.M., Zraika S., Udayasankar J., McNeely M.J., Carr D.B., Leonetti D.L., Young B.A., de Boer I.H. (2009). Body mass index is associated with increased creatinine clearance by a mechanism independent of body fat distribution. J. Clin. Endocrinol. Metab..

[11] Foster M.C., Hwang S.J., Larson M.G., Lichtman J.H., Parikh N.I., Vasan R.S., Levy D., Fox C.S. (2008). Overweight, obesity, and the development of stage 3 CKD: The framingham heart study. Am. J. Kidney Dis..

[12] Oh H., Quan S.A., Jeong J.Y., Jang S.N., Lee J.E., Kim D.H. (2013). Waist circumference, not body mass index, is associated with renal function decline in korean population: Hallym aging study. PLoS ONE.

[13] Noori N., Hosseinpanah F., Nasiri A.A., Azizi F. (2009). Comparison of overall obesity and abdominal adiposity in predicting chronic kidney disease incidence among adults. J. Ren. Nutr..

[14] Burton J.O., Gray L.J., Webb D.R., Davies M.J., Khunti K., Crasto W., Carr S.J., Brunskill N.J. (2012). Association of anthropometric obesity measures with chronic kidney disease risk in a non-diabetic patient population. Nephrol. Dial. Transplant..

[15] Liu J., Chen Z., Li W., Xu G., Liu J., Yi B., Mao J., Huang J., Yang S., Zhang H. (2016). Obesity indices for prediction of chronic kidney disease: A cross-sectional study in 26,655 Chinese adults. J. Centr. South Univ. Med. Sci..

[16] Sakurai M., Kobayashi J., Takeda Y., Nagasawa S.Y., Yamakawa J., Moriya J., Mabuchi H., Nakagawa H. (2016). Sex differences in associations among obesity, metabolic abnormalities, and chronic kidney disease in Japanese men and women. J. Epidemiol. Jpn. Epidemiol. Assoc..

[17] Huang J.C., Lin H.Y., Lim L.M., Chen S.C., Chang J.M., Hwang S.J., Tsai J.C., Hung C.C., Chen H.C. (2015). Body mass index, mortality, and gender difference in advanced chronic kidney disease. PLoS ONE.

[18] Wang Z., Zeng X., Chen Z., Wang X., Zhang L., Zhu M., Yi D. (2015). Association of visceral and total body fat with hypertension and prehypertension in a middle-aged Chinese population. J. Hypertens..

[19] Abbasi S.A., Hundley W.G., Bluemke D.A., Jerosch-Herold M., Blankstein R., Petersen S.E., Rider O.J., Lima J.A., Allison M.A., Murthy V.L. (2015). Visceral adiposity and left ventricular remodeling: The Multi-Ethnic study of atherosclerosis. Nutr. Metab. Cardiovasc. Dis..

[20] Yuan M., Hsu F.C., Bowden D.W., Xu J., Carrie Smith S., Wagenknecht L.E., Comeau M.E., Divers J., Register T.C., Jeffrey Carr J. (2016). Relationships between measures of adiposity with subclinical atherosclerosis in patients with type 2 diabetes. Obesity.

[21] Xu X., Zhao Y., Zhao Z., Zhu S., Liu X., Zhou C., Shao X., Liang Y., Duan C., Holthofer H. (2016). Correlation of visceral adiposity index with chronic kidney disease in the People‘s Republic of China: To rediscover the new clinical potential of an old indicator for visceral obesity. Ther. Clin. Risk Manag..

[22] Thompson A.L., Adair L., Gordon-Larsen P., Zhang B., Popkin B. (2015). Environmental, dietary, and behavioral factors distinguish chinese adults with high waist-to-height ratio with and without inflammation. J. Nutr..

[23] Bozorgmanesh M., Hadaegh F., Azizi F. (2010). Diabetes prediction, lipid accumulation product, and adiposity measures; 6-year follow-up: Tehran lipid and glucose study. Lipids Health Dis..

[24] Bozorgmanesh M., Hadaegh F., Azizi F. (2010). Predictive performances of lipid accumulation product vs. adiposity measures for cardiovascular diseases and all-cause mortality, 8.6-year follow-up: Tehran lipid and glucose study. Lipids Health Dis..

[25] Ioachimescu A.G., Brennan D.M., Hoar B.M., Hoogwerf B.J. (2010). The lipid accumulation product and all-cause mortality in patients at high cardiovascular risk: A PreCIS database study. Obesity.

[26] Kahn H.S. (2005). The “lipid accumulation product“ performs better than the body mass index for recognizing cardiovascular risk: A population-based comparison. BioMed. Centr..

[27] Liu X., Wang Y., Wang C., Shi C., Cheng C., Chen J., Ma H., Lv L., Li L., Lou T. (2013). A new equation to estimate glomerular filtration rate in Chinese elderly population. PLoS ONE.

[28] Amato M.C., Giordano C., Galia M., Criscimanna A., Vitabile S., Midiri M., Galluzzo A. (2010). Visceral Adiposity Index: A reliable indicator of visceral fat function associated with cardiometabolic risk. Diabet. Care.

[29] Ma Y.C., Zuo L., Chen J.H., Luo Q., Yu X.Q., Li Y., Xu J.S., Huang S.M., Wang L.N., Huang W. (2006). Modified glomerular filtration rate estimating equation for Chinese patients with chronic kidney disease. J. Am. Soc. Nephrol..

[30] Shively C.A., Register T.C., Clarkson T.B. (2009). Social stress, visceral obesity, and coronary artery atherosclerosis: Product of a primate adaptation. Am. J. Primatol..

[31] Henegar J.R., Bigler S.A., Henegar L.K., Tyagi S.C., Hall J.E. (2001). Functional and structural changes in the kidney in the early stages of obesity. J. Am. Soc. Nephrol..

[32] Sharma K. (2014). Obesity, oxidative stress, and fibrosis in chronic kidney disease. Kidney Int. Suppl..

[33] Stefan N., Artunc F., Heyne N., Machann J., Schleicher E.D., Haring H.U. (2016). Obesity and renal disease: Not all fat is created equal and not all obesity is harmful to the kidneys. Nephrol. Dial. Transplant..

[34] Huang J., Zhou C., Li Y., Zhu S., Liu A., Shao X., Liu X., Holthfer H., Zou H. (2015). Visceral adiposity index, hypertriglyceridemic waist phenotype and chronic kidney disease in a southern Chinese population: A cross-sectional study. Int. Urol. Nephrol..

[35] Tripathy J.P., Thakur J.S., Jeet G., Chawla S., Jain S., Prasad R. (2016). Urban rural differences in diet, physical activity and obesity in India: Are we witnessing the great Indian equalisation? Results from a cross-sectional STEPS survey. BMC Public Health.

[36] Palmer B.F., Clegg D.J. (2015). The sexual dimorphism of obesity. Mol. Cell. Endocrinol..

[37] Ikeda M., Swide T., Vayl A., Lahm T., Anderson S., Hutchens M.P. (2015). Estrogen administered after cardiac arrest and cardiopulmonary resuscitation ameliorates acute kidney injury in a sex- and age-specific manner. Crit. Care.

[38] Cornier M.A., Despres J.P., Davis N., Grossniklaus D.A., Klein S., Lamarche B., Lopez-Jimenez F., Rao G., St-Onge M.P., Towfighi A. (2011). Assessing adiposity: A scientific statement from the American Heart Association. Circulation.

[39] Du T., Yu X., Zhang J., Sun X. (2015). Lipid accumulation product and visceral adiposity index are effective markers for identifying the metabolically obese normal-weight phenotype. Acta Diabetol..

[40] Zhang X.H., Zhang M., He J., Yan Y.Z., Ma J.L., Wang K., Ma R.L., Guo H., Mu L.T., Ding Y.S. (2016). Comparison of anthropometric and atherogenic indices as screening tools of metabolic syndrome in the Kazakh adult population in Xinjiang. Int. J. Environ. Res. Public Health.

[41] Zhang L., Wang F., Wang L., Wang W., Liu B., Liu J., Chen M., He Q., Liao Y., Yu X. (2012). Prevalence of chronic kidney disease in China: A cross-sectional survey. Lancet.

